# (2*E*)-2-(Furan-2-yl­methyl­idene)-2,3-dihydro-1*H*-inden-1-one

**DOI:** 10.1107/S1600536812010501

**Published:** 2012-03-14

**Authors:** Abdullah M. Asiri, Hassan M. Faidallah, Khulud F. Al-Nemari, Seik Weng Ng, Edward R. T. Tiekink

**Affiliations:** a Chemistry Department, Faculty of Science, King Abdulaziz University, PO Box 80203, Jeddah, Saudi Arabia; b The Center of Excellence for Advanced Materials Research, King Abdulaziz University, Jeddah, PO Box 80203, Saudi Arabia; c Department of Chemistry, University of Malaya, 50603 Kuala Lumpur, Malaysia

## Abstract

In the title compound, C_14_H_10_O_2_, the five-membered ring of the inden-1-one residue is almost planar (r.m.s. deviation = 0.035 Å). A twist about the single bond linking the two residues is evident [C—C—C—C torsion angle = −13.2 (5)°]. The three-dimensional architecture is stabilized by C—H⋯O (involving the trifurcated carbonyl O atom), C—H⋯π and π–π inter­actions [between the five- and six-membered rings of inden-1-one residues; ring centroid–centroid distance = 3.7983 (17) Å]. The sample studied was a non-merohedral twin; the minor component refined to approximately 36%.

## Related literature

For the biological activity of related species, see: Vera-DiVaio *et al.* (2009 ▶). For related structures, see: Asiri *et al.* (2012*a* ▶,*b* ▶). For the treatment of twinned data, see: Spek (2009 ▶).
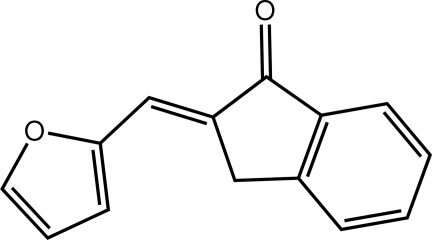


## Experimental

### 

#### Crystal data

C_14_H_10_O_2_
*M* *_r_* = 210.22
Monoclinic, 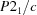

*a* = 5.9333 (8) Å
*b* = 7.6605 (6) Å
*c* = 22.386 (3) Å
β = 91.582 (14)°
*V* = 1017.1 (2) Å^3^
*Z* = 4
Mo *K*α radiation
μ = 0.09 mm^−1^
*T* = 100 K
0.25 × 0.25 × 0.05 mm

#### Data collection

Agilent SuperNova Dual diffractometer with an Atlas detector
Absorption correction: multi-scan (*CrysAlis PRO*; Agilent, 2011 ▶) *T*_min_ = 0.978, *T*_max_ = 0.995
5052 measured reflections
3274 independent reflections
2683 reflections with *I* > 2σ(*I*)
*R*_int_ = 0.086

#### Refinement

*R*[*F* ^2^ > 2σ(*F* ^2^)] = 0.081
*wR*(*F* ^2^) = 0.251
*S* = 1.10
3274 reflections
146 parameters
H-atom parameters constrained
Δρ_max_ = 0.49 e Å^−3^
Δρ_min_ = −0.38 e Å^−3^

### 

Data collection: *CrysAlis PRO* (Agilent, 2011 ▶); cell refinement: *CrysAlis PRO*; data reduction: *CrysAlis PRO*; program(s) used to solve structure: *SHELXS97* (Sheldrick, 2008 ▶); program(s) used to refine structure: *SHELXL97* (Sheldrick, 2008 ▶); molecular graphics: *ORTEP-3* (Farrugia, 1997 ▶) and *DIAMOND* (Brandenburg, 2006 ▶); software used to prepare material for publication: *publCIF* (Westrip, 2010 ▶).

## Supplementary Material

Crystal structure: contains datablock(s) global, I. DOI: 10.1107/S1600536812010501/bt5838sup1.cif

Structure factors: contains datablock(s) I. DOI: 10.1107/S1600536812010501/bt5838Isup2.hkl

Supplementary material file. DOI: 10.1107/S1600536812010501/bt5838Isup3.cml

Additional supplementary materials:  crystallographic information; 3D view; checkCIF report

## Figures and Tables

**Table 1:** Hydrogen-bond geometry (Å, °) *Cg*1 is the centroid of the C2–C7 ring.

*D*—H⋯*A*	*D*—H	H⋯*A*	*D*⋯*A*	*D*—H⋯*A*
C3—H3⋯O1^i^	0.95	2.56	3.414 (4)	149
C8—H8*A*⋯O1^ii^	0.99	2.37	3.343 (4)	166
C14—H14⋯O1^iii^	0.95	2.45	3.372 (4)	164
C8—H8*B*⋯*Cg*1^iv^	0.99	2.70	3.517 (3)	140

Symmetry codes: (i) 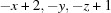; (ii) 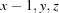; (iii) 
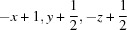
; (iv) 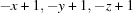.

**Fig. 1.**
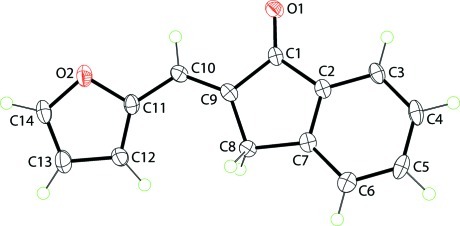
The molecular structure of (I) showing the atom-labelling scheme and displacement ellipsoids at the 50% probability level.

**Fig. 2.**
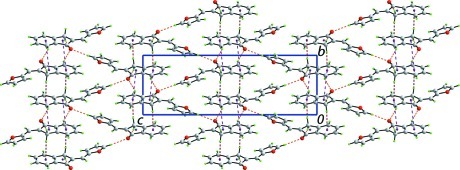
A view in projection down the a axis of the unit-cell contents of (I). The C—H···O, C—H···π and π–π interactions are shown as orange, brown and purple dashed lines, respectively.

## supplementary crystallographic information

### Comment

The title compound, 2-furan-2-ylmethylene-indan-1-one (I), has been investigated crystallographically in connection with recent structural studies on related derivatives (Asiri *et al.*, 2012*a*; Asiri *et al.*, 2012*b*). The motivation for the original synthesis of (I) is its relationship to biologically active compounds (Vera-DiVaio *et al.*, 2009).

In the molecule of (I), Fig. 1, the five-membered ring of the inden-1-one residue is planar with the r.m.s. deviation for the five atoms = 0.035 Å [maximum deviations = 0.031 (2) for the C9 atom and -0.026 (3) for the C8 atom]. A twist in the molecule about the C10—C11 bond is evident with the C9—C10—C11—C12 torsion angle being -13.2 (5)°. The configuration about the C9═C10 bond [1.346 (4) Å] is *E*.

The carbonyl-O1 atom is tri-furcated, forming three C—H···O interactions which lead to a three-dimensional architecture. Additional interactions in the crystal packing include C—H···π interactions, Table 1, as well as π–π contacts between the five- and six-membered rings of inden-1-one residue [ring centroid···centroid distance = 3.7983 (17) Å, angle of inclination = 1.02 (14)° for symmetry operation 1 - *x*, -*y*, 1 - *z*], Fig. 2.

### Experimental

A solution of the furan-2-carboxaldehyde (0.95 g, 0.01 *M*) in ethanol (20 ml) was added to a stirred solution of 1-indanone (1.3 g,0.01 *M*) in (20%) ethanolic KOH (20 ml), and stirring was maintained at room temperature for 6 h. The reaction mixture was then poured into water (200 ml) and set aside overnight. The precipitated solid product was collected by filtration, washed with water, dried and recrystallized from ethanol. Yield: 92%. *M*.pt: 393–395 K.

### Refinement

Carbon-bound H-atoms were placed in calculated positions [C—H = 0.95 to 0.99 Å, *U*_iso_(H) = 1.2*U*_eq_(C)] and were included in the refinement in the riding model approximation.

The studied sample was a non-merohedral twin (the twin law is 1 0 0.015, 0 1 0, 0 0 1). The twin domains were separated by using the *TwinRotMat* routine in PLATON (Spek, 2009) and the minor component refined to 0.362 (3).

### Crystal data

**Table:**

C_14_H_10_O_2_	*F*(000) = 440
*M**_r_* = 210.22	*D*_x_ = 1.373 Mg m^−^^3^
Monoclinic, *P*2_1_/*c*	Mo *K*α radiation, λ = 0.71073 Å
Hall symbol: -P 2ybc	Cell parameters from 1169 reflections
*a* = 5.9333 (8) Å	θ = 2.7–27.5°
*b* = 7.6605 (6) Å	µ = 0.09 mm^−^^1^
*c* = 22.386 (3) Å	*T* = 100 K
β = 91.582 (14)°	Plate, light-brown
*V* = 1017.1 (2) Å^3^	0.25 × 0.25 × 0.05 mm
*Z* = 4	

### Data collection

**Table:**

Agilent SuperNova Dual diffractometer with an Atlas detector	3274 independent reflections
Radiation source: SuperNova (Mo) X-ray Source	2683 reflections with *I* > 2σ(*I*)
Mirror monochromator	*R*_int_ = 0.086
Detector resolution: 10.4041 pixels mm^-1^	θ_max_ = 27.6°, θ_min_ = 2.8°
ω scan	*h* = −7→7
Absorption correction: multi-scan (*CrysAlis PRO*; Agilent, 2011)	*k* = −9→9
*T*_min_ = 0.978, *T*_max_ = 0.995	*l* = −28→29
5052 measured reflections	

### Refinement

**Table:**

Refinement on *F*^2^	Primary atom site location: structure-invariant direct methods
Least-squares matrix: full	Secondary atom site location: difference Fourier map
*R*[*F*^2^ > 2σ(*F*^2^)] = 0.081	Hydrogen site location: inferred from neighbouring sites
*wR*(*F*^2^) = 0.251	H-atom parameters constrained
*S* = 1.10	*w* = 1/[σ^2^(*F*_o_^2^) + (0.1697*P*)^2^ + 0.358*P*] where *P* = (*F*_o_^2^ + 2*F*_c_^2^)/3
3274 reflections	(Δ/σ)_max_ = 0.001
146 parameters	Δρ_max_ = 0.49 e Å^−^^3^
0 restraints	Δρ_min_ = −0.38 e Å^−^^3^

### Fractional atomic coordinates and isotropic or equivalent isotropic displacement parameters (Å^2^)

**Table:**

	*x*	*y*	*z*	*U*_iso_*/*U*_eq_	
O1	0.8036 (3)	0.1044 (3)	0.41473 (10)	0.0225 (5)	
O2	0.3114 (4)	0.3830 (3)	0.25105 (10)	0.0247 (6)	
C1	0.6426 (5)	0.1749 (3)	0.43739 (13)	0.0158 (6)	
C2	0.6084 (5)	0.2043 (3)	0.50122 (13)	0.0165 (6)	
C3	0.7489 (5)	0.1602 (4)	0.55034 (14)	0.0198 (6)	
H3	0.8888	0.1028	0.5449	0.024*	
C4	0.6783 (5)	0.2028 (4)	0.60685 (14)	0.0248 (7)	
H4	0.7724	0.1762	0.6406	0.030*	
C5	0.4707 (6)	0.2842 (4)	0.61507 (14)	0.0249 (7)	
H5	0.4248	0.3100	0.6544	0.030*	
C6	0.3307 (5)	0.3279 (4)	0.56689 (14)	0.0217 (7)	
H6	0.1902	0.3840	0.5727	0.026*	
C7	0.4012 (5)	0.2875 (3)	0.50936 (14)	0.0174 (6)	
C8	0.2794 (5)	0.3192 (4)	0.44983 (13)	0.0173 (6)	
H8A	0.1365	0.2526	0.4469	0.021*	
H8B	0.2464	0.4448	0.4439	0.021*	
C9	0.4447 (5)	0.2549 (3)	0.40530 (14)	0.0171 (6)	
C10	0.4384 (5)	0.2705 (4)	0.34539 (14)	0.0176 (6)	
H10	0.5628	0.2246	0.3247	0.021*	
C11	0.2621 (5)	0.3496 (4)	0.31001 (14)	0.0195 (6)	
C12	0.0463 (5)	0.4025 (4)	0.31992 (15)	0.0237 (7)	
H12	−0.0304	0.3951	0.3565	0.028*	
C13	−0.0413 (6)	0.4706 (4)	0.26501 (16)	0.0274 (8)	
H13	−0.1881	0.5165	0.2576	0.033*	
C14	0.1252 (5)	0.4571 (4)	0.22545 (15)	0.0248 (7)	
H14	0.1135	0.4946	0.1850	0.030*	

### Atomic displacement parameters (Å^2^)

**Table:**

	*U*^11^	*U*^22^	*U*^33^	*U*^12^	*U*^13^	*U*^23^
O1	0.0194 (11)	0.0244 (11)	0.0235 (12)	0.0027 (9)	−0.0027 (9)	−0.0039 (9)
O2	0.0255 (12)	0.0295 (11)	0.0186 (11)	−0.0028 (10)	−0.0054 (9)	0.0042 (9)
C1	0.0176 (14)	0.0118 (12)	0.0178 (14)	−0.0041 (11)	−0.0043 (11)	0.0015 (11)
C2	0.0193 (15)	0.0099 (12)	0.0200 (15)	−0.0036 (11)	−0.0035 (11)	0.0008 (11)
C3	0.0190 (14)	0.0170 (13)	0.0229 (15)	−0.0047 (12)	−0.0074 (11)	0.0033 (12)
C4	0.0303 (18)	0.0219 (15)	0.0217 (16)	−0.0050 (13)	−0.0100 (13)	0.0099 (12)
C5	0.0370 (19)	0.0245 (15)	0.0131 (14)	−0.0084 (14)	0.0009 (13)	0.0016 (12)
C6	0.0230 (16)	0.0201 (14)	0.0221 (15)	−0.0078 (13)	0.0014 (12)	0.0004 (12)
C7	0.0208 (15)	0.0106 (11)	0.0205 (15)	−0.0046 (11)	−0.0030 (11)	−0.0005 (11)
C8	0.0172 (15)	0.0148 (12)	0.0197 (14)	−0.0006 (11)	−0.0011 (11)	−0.0028 (11)
C9	0.0149 (14)	0.0127 (12)	0.0233 (15)	−0.0041 (11)	−0.0037 (11)	−0.0002 (12)
C10	0.0151 (14)	0.0166 (13)	0.0211 (15)	−0.0026 (11)	−0.0029 (11)	−0.0033 (11)
C11	0.0256 (15)	0.0161 (13)	0.0166 (15)	0.0000 (12)	−0.0046 (12)	−0.0015 (11)
C12	0.0223 (16)	0.0281 (15)	0.0204 (16)	0.0048 (13)	−0.0033 (11)	−0.0011 (13)
C13	0.032 (2)	0.0290 (16)	0.0210 (16)	0.0024 (15)	−0.0080 (12)	0.0009 (14)
C14	0.0253 (18)	0.0261 (15)	0.0224 (17)	−0.0044 (13)	−0.0097 (12)	0.0065 (13)

### Geometric parameters (Å, º)

**Table:**

O1—C1	1.220 (4)	C6—H6	0.9500
O2—C14	1.355 (4)	C7—C8	1.518 (4)
O2—C11	1.384 (4)	C8—C9	1.501 (4)
C1—C2	1.466 (4)	C8—H8A	0.9900
C1—C9	1.491 (4)	C8—H8B	0.9900
C2—C7	1.401 (4)	C9—C10	1.346 (4)
C2—C3	1.403 (4)	C10—C11	1.429 (4)
C3—C4	1.383 (5)	C10—H10	0.9500
C3—H3	0.9500	C11—C12	1.367 (4)
C4—C5	1.398 (5)	C12—C13	1.420 (4)
C4—H4	0.9500	C12—H12	0.9500
C5—C6	1.384 (4)	C13—C14	1.349 (5)
C5—H5	0.9500	C13—H13	0.9500
C6—C7	1.400 (5)	C14—H14	0.9500
			
C14—O2—C11	106.8 (2)	C9—C8—H8A	111.2
O1—C1—C2	127.2 (3)	C7—C8—H8A	111.2
O1—C1—C9	126.6 (3)	C9—C8—H8B	111.2
C2—C1—C9	106.1 (2)	C7—C8—H8B	111.2
C7—C2—C3	120.8 (3)	H8A—C8—H8B	109.1
C7—C2—C1	110.0 (3)	C10—C9—C1	121.1 (3)
C3—C2—C1	129.2 (3)	C10—C9—C8	129.2 (3)
C4—C3—C2	118.2 (3)	C1—C9—C8	109.6 (2)
C4—C3—H3	120.9	C9—C10—C11	126.1 (3)
C2—C3—H3	120.9	C9—C10—H10	116.9
C3—C4—C5	121.1 (3)	C11—C10—H10	116.9
C3—C4—H4	119.5	C12—C11—O2	108.9 (3)
C5—C4—H4	119.5	C12—C11—C10	135.2 (3)
C6—C5—C4	121.2 (3)	O2—C11—C10	115.9 (3)
C6—C5—H5	119.4	C11—C12—C13	106.9 (3)
C4—C5—H5	119.4	C11—C12—H12	126.6
C5—C6—C7	118.4 (3)	C13—C12—H12	126.6
C5—C6—H6	120.8	C14—C13—C12	106.4 (3)
C7—C6—H6	120.8	C14—C13—H13	126.8
C6—C7—C2	120.4 (3)	C12—C13—H13	126.8
C6—C7—C8	128.7 (3)	C13—C14—O2	111.0 (3)
C2—C7—C8	110.9 (3)	C13—C14—H14	124.5
C9—C8—C7	103.1 (2)	O2—C14—H14	124.5
			
O1—C1—C2—C7	−179.3 (3)	O1—C1—C9—C10	−6.6 (4)
C9—C1—C2—C7	2.9 (3)	C2—C1—C9—C10	171.3 (3)
O1—C1—C2—C3	0.2 (5)	O1—C1—C9—C8	177.2 (3)
C9—C1—C2—C3	−177.6 (3)	C2—C1—C9—C8	−5.0 (3)
C7—C2—C3—C4	−0.6 (4)	C7—C8—C9—C10	−170.9 (3)
C1—C2—C3—C4	179.9 (3)	C7—C8—C9—C1	5.0 (3)
C2—C3—C4—C5	1.3 (4)	C1—C9—C10—C11	−177.8 (3)
C3—C4—C5—C6	−1.2 (5)	C8—C9—C10—C11	−2.4 (5)
C4—C5—C6—C7	0.4 (4)	C14—O2—C11—C12	0.4 (3)
C5—C6—C7—C2	0.2 (4)	C14—O2—C11—C10	180.0 (2)
C5—C6—C7—C8	179.2 (3)	C9—C10—C11—C12	−13.2 (5)
C3—C2—C7—C6	−0.1 (4)	C9—C10—C11—O2	167.3 (3)
C1—C2—C7—C6	179.5 (2)	O2—C11—C12—C13	0.2 (3)
C3—C2—C7—C8	−179.3 (2)	C10—C11—C12—C13	−179.4 (3)
C1—C2—C7—C8	0.3 (3)	C11—C12—C13—C14	−0.6 (4)
C6—C7—C8—C9	177.6 (3)	C12—C13—C14—O2	0.9 (4)
C2—C7—C8—C9	−3.3 (3)	C11—O2—C14—C13	−0.8 (3)

### Hydrogen-bond geometry (Å, º)

Cg1 is the centroid of the C2–C7 ring.

**Table:**

*D*—H···*A*	*D*—H	H···*A*	*D*···*A*	*D*—H···*A*
C3—H3···O1^i^	0.95	2.56	3.414 (4)	149
C8—H8*A*···O1^ii^	0.99	2.37	3.343 (4)	166
C14—H14···O1^iii^	0.95	2.45	3.372 (4)	164
C8—H8*B*···*Cg*1^iv^	0.99	2.70	3.517 (3)	140

Symmetry codes: (i) −*x*+2, −*y*, −*z*+1; (ii) *x*−1, *y*, *z*; (iii) −*x*+1, *y*+1/2, −*z*+1/2; (iv) −*x*+1, −*y*+1, −*z*+1.
